# Machine-learning classification of texture features of portable chest X-ray accurately classifies COVID-19 lung infection

**DOI:** 10.1186/s12938-020-00831-x

**Published:** 2020-11-25

**Authors:** Lal Hussain, Tony Nguyen, Haifang Li, Adeel A. Abbasi, Kashif J. Lone, Zirun Zhao, Mahnoor Zaib, Anne Chen, Tim Q. Duong

**Affiliations:** 1 Department of Computer Science and IT, King Abdullah Campus, University of Azad Jammu and Kashmir, Muzaffarabad, 13100 Azad Kashmir Pakistan; 2 Department of Computer Science and IT, Neelum Campus, University of Azad Jammu and Kashmir, Athmuqam, 13230 Azad Kashmir Pakistan; 3 Department of Radiology, Renaissance School of Medicine at Stony Brook University, 101 Nicolls Rd, Stony Brook, NY 11794 USA

**Keywords:** Texture, Morphological, Machine learning, Feature extraction, Classification, COVID-19

## Abstract

**Background:**

The large volume and suboptimal image quality of portable chest X-rays (CXRs) as a result of the COVID-19 pandemic could post significant challenges for radiologists and frontline physicians. Deep-learning artificial intelligent (AI) methods have the potential to help improve diagnostic efficiency and accuracy for reading portable CXRs.

**Purpose:**

The study aimed at developing an AI imaging analysis tool to classify COVID-19 lung infection based on portable CXRs.

**Materials and methods:**

Public datasets of COVID-19 (*N* = 130), bacterial pneumonia (*N* = 145), non-COVID-19 viral pneumonia (*N* = 145), and normal (*N* = 138) CXRs were analyzed. Texture and morphological features were extracted. Five supervised machine-learning AI algorithms were used to classify COVID-19 from other conditions. Two-class and multi-class classification were performed. Statistical analysis was done using unpaired two-tailed *t* tests with unequal variance between groups. Performance of classification models used the receiver-operating characteristic (ROC) curve analysis.

**Results:**

For the two-class classification, the accuracy, sensitivity and specificity were, respectively, 100%, 100%, and 100% for COVID-19 vs normal; 96.34%, 95.35% and 97.44% for COVID-19 vs bacterial pneumonia; and 97.56%, 97.44% and 97.67% for COVID-19 vs non-COVID-19 viral pneumonia. For the multi-class classification, the combined accuracy and AUC were 79.52% and 0.87, respectively.

**Conclusion:**

AI classification of texture and morphological features of portable CXRs accurately distinguishes COVID-19 lung infection in patients in multi-class datasets. Deep-learning methods have the potential to improve diagnostic efficiency and accuracy for portable CXRs.

## Background

In December 2019, in the Wuhan Hubei province of China, a cluster of cases of pneumonia with an unknown cause was reported [1]. Eventually, it was discovered as severe acute respiratory syndrome coronavirus-2 (SARS-CoV-2, previously named as 2019 novel coronavirus or COVID-19) which has then caused major public health issues and became a large global outbreak. According to the recent statistics, there are millions of confirmed cases in United States and India, and the number is still increasing. The WHO also declared on January 13, 2020 that COVID-19 was the sixth public health emergency of international concern following H1N1 (2009), polio (2014), Ebola in West Africa (2014), Zika (2016) and Ebola in the Democratic Republic of Congo (2019) [2]. It was also found that the novel coronaviral pneumonia is similar to another severe acute respiratory syndrome caused by the Middle East respiratory syndrome (MERS) coronavirus and that it was also capable of causing a more severe form known as acute respiratory distress syndrome (ARDS) [3, 4]. Consensus, criteria, and guidelines were being established with the aim to prevent transmission and facilitate diagnosis and treatment [2, 5, 6]. The rapid incidences of infection are due in part by the relatively slow onset of symptoms, thus enabling widespread transmission by asymptomatic carriers [7]. Along with the global connectivity of today’s travel society, this infection readily spread worldwide [7], giving rise to a pandemic [8, 9].

Radiological imaging of the COVID-19 pneumonia reveals the destruction of pulmonary parenchyma which includes extensive consolidation and interstitial inflammation as previously reported in other coronavirus infections [10, 11]. In total, interstitial lung disease (ILD) comprises of more than 200 different types of chronic lung disorders that is characterized by inflammation of lung tissue, usually referred to as pulmonary fibrosis. The fibrosis causes lung stiffness, and this reduces the ability of the air sacs (i.e., spaces within an organism where there is the constant presence of air) to carry out and deliver oxygen into the bloodstream. This eventually can lead to the permanent loss of the ability to breathe. The ILDs are also heterogeneous diseases histologically but mostly contain similar clinical manifestations to each other or with other different lung disorders. This makes determining the differential diagnosis difficult. In addition, the large quantity of radiological data that radiologists are required to scrutinize (with lack of strict clinical guidelines) leads to a low diagnostic accuracy and high inter- and intra-observer variability, which was reported as great as 50% [12].

The most commonly used diagnosis for COVID-19 infections is through reverse transcription-polymerase chain reaction (RT-PCR) assays of nasopharyngeal swabs [13]. However, the high false-negative rate [14], length of test, and shortage of RT-PCR assay kits for the early stages of the outbreak can restrict a prompt diagnosis of infected patients. Computed tomography (CT) and chest X-ray (CXR) are well suited to image the lung of COVID-19 infections. In contrast to the swab test, CT and CXR reveals a spatial location of the suspected pathology as well as the extent of damages. The hallmark pathology of CXR are bilateral distribution of peripheral hazy lung opacities include air space consolidation [15]. The advantage of imaging is that it has good sensitivity, a fast turnaround time, and it can visualize the extent of infection in the lung. The disadvantage of imaging is that it has low specificity, challenging to distinguish different types of lung infection especially when there is severity in the lung infection.

Computer-aided diagnostic (CAD) systems can assist radiologists to increase diagnostic accuracy. Currently, researchers are using the hand-crafted or learning features which are based on the texture, geometry, and morphological characteristics of the lung for detection. However, it is often crucial and challenging to choose the appropriate classifier that can optimally handle the property of the feature spaces of the lung. The traditional image recognition methods are Bayesian networks (BNs), support vector machine (SVM), artificial neural networks (ANNs), k-nearest neighbors (kNN), and Adaboost, decision trees (DTs). These machine-learning methods [16, 17] require hand-crafted features to compute such as texture, SIFT, entropy, morphological, elliptic Fourier descriptors (EFDs), shape, geometry, density of pixels, and off-shelf classifiers as explained in [18]. In addition, the machine-learning (ML) feature-based methods are known as non-deep learning methods. There are many applications for these non-deep learning methods such as uses in neurodegenerative diseases, cancer detection, and psychiatric diseases. [17, 19–22]. However, the major limitations of non-deep learning methods are that they are dependent on the feature extraction step and this makes it difficult to find the most relevant feature which are needed to obtain the most effective result. To overcome these difficulties, the use of artificial intelligence (AI) can be employed. The AI technology in the field of medical imaging is becoming popular especially for the technology advancement and development of deep learning [23–32]. Recently, [33] used Inf-net for automatic detection of COVID-19 lung infection segmentation from CT images. Moreover, [18] employed momentum contrastive learning for few shot COVID-19 diagnosis from chest CT images. There are vast applications of deep convolutional neural network (DCNN) and machine-learning algorithms in medical imaging problems [32, 34–38]; however, this study is specifically aimed to apply machine-learning algorithms with feature extraction approach. The main advantage of this method is the ability to learn the adaptive image features and classification, which are able to be performed simultaneously. The general goals are to develop automated tools by employing and optimizing machine-learning models along with texture and morphological features to detect early, to distinguish coronavirus-infected patients from non-infected patients. This proposed method will help the healthcare clinicians and radiologists for further diagnosis and tracking the disease progression. The AI-based system, once verified, and tested can lead towards crucial detection and control of patients affected from COVID-19. Furthermore, the machine-learning image analysis tools can potentially support the radiologists by providing an initial read or second opinion.

In this study, we employed machine-learning methods to classify texture features of portable CXRs with the aim to identify COVID-19 lung infection. Comparison of texture and morphological features on COVID-19, bacterial pneumonia, non-COVID-19 viral pneumonia, and normal CXRs were made. AI-based classification methods were used for differential diagnosis of COVID-19 lung infection. We tested the hypothesis that AI classification of texture features of CXR can accurately detect the COVID-19 lung infection.

## Results

We applied five supervised machine-learning classifiers (XGB-L, XGB-Tree, CART, KNN and Naïve Bayes) to classify COVID-19 from bacterial pneumonia, non-COVID-19 viral pneumonia, and normal lung CXRs.

Table 1 shows the results of AI classification of texture and morphological features for COVID-19 vs normal utilizing five different classifiers: XGB-L, XGB-Tree, CART (DT), KNN, and Naïve Bayes. All classifiers yielded essentially 100% accuracy by all performance measures along with top four ranked features (i.e., compactness, thin ratio, perimeter, standard deviation), indicating that there is significant difference between the two groups.

**Table 1:** Performance of AI classification of texture and morphological features utilizing five different classifiers of COVID-19 (*N* = 130) vs normal (*N* = 138)

Classifier	Sensitivity (%)	Specificity (%)	PPV (%)	NPV (%)	Accuracy (%)	AUC (LB, UP)	*p* value
XGB-L	100	100	100	100	100	1.00	2.00e−16
XGB-Tree	100	100	1003	100	100	1.00	2.00e−16
CART (DT)	100	100	100	100	100	1.00	2.00e−16
KNN	89.71	100	100	91.11	95	0.99	2.00e−16
Naïve Bayes	100	100	100	100	100	1.00	2.00e−16

Table 2 shows the results of AI classification of texture and morphological features for COVID-19 vs bacterial pneumonia. All classifiers except KNN performed well by all performance measures. Specifically, the XGB-L and XGB-Tree classifier yielded the highest classification accuracy (96.34% and 91.46%, respectively), while KNN classifier performed the worst (accuracy of 71.95%). While with the top four ranking features, the XGB-L and XGB-tree classifiers yielded highest accuracy of 85.37% and 86.59%, respectively.

**Table 2:** Performance of AI classification of texture and morphological features utilizing five different classifiers of COVID-19 (*N* = 130) vs bacterial pneumonia (*N* = 145)

Classifier	Sensitivity (%)	Specificity (%)	PPV (%)	NPV (%)	Accuracy (%)	AUC (LB, UP)	*p* value
XGB-L	95.35	97.44	97.62	95.00	96.34	0.98 (0.96, 1.00)	2.19e−14
XGB-Tree	88.37	94.87	95.00	88.10	91.46	0.97 (0.93, 1.00)	2.19e−14
CART (DT)	74.42	97.44	96.97	77.55	85.37	0.92 (0.87, 0.98)	3.22e−10
KNN	86.05	56.41	68.52	78.57	71.95	0.83 (0.74, 0.92)	2.40e−04
Naïve Bayes	90.70	79.49	82.98	88.51	85.37	0.92 (0.85, 0.98)	3.22e−10

Table 3 shows the results of AI classification of texture and morphological features for COVID-19 vs non-COVID viral pneumonia. All classifiers except KNN performed well by all performance measures. Specifically, the XGB-L and XGB-Tree classifier yielded the highest classification accuracy (97.56% and 95.12%, respectively), while KNN classifier performed the worst (accuracy of 79.27%).

**Table 3:** Performance of AI classification of texture and morphological features utilizing five different classifiers of COVID-19 (*N* = 130) vs viral pneumonia (*N* = 145)

Classifier	Sensitivity	Specificity	PPV	NPV	Accuracy	AUC (LB, UP)	*p* value
XGB-L	97.44	97.67	97.44	97.67	97.56	0.98 (0.96, 1.00)	2.00e−16
XGB-Tree	94.87	95.35	94.87	94.87	95.12	0.98 (0.95, 1.00)	2.00e−16
CART (DT)	94.87	93.02	92.50	95.24	93.90	0.94 (0.89, 0.99)	2.00e−16
KNN	69.63	88.37	84.38	76.00	79.27	0.85 (0.77, 0.94)	4.38e−07
Naïve Bayes	64.10	95.35	92.59	74.55	80.49	0.93 (0.88, 0.98)	1.20e−07

Table 4 shows the two-class classification using the XGB-L classifier. The result showed that model classified COVID-19 from normal patients most accurately, followed by COVID-19 from bacterial pneumonia, and lastly by COVID-19 from viral pneumonia.

**Table 4:** Two-class classification using XGB-linear with texture + morphological features for COVID-19 (*N* = 130) vs bacterial pneumonia (*N* = 145), COVID-19 vs non-COVID-19 viral (*N* = 145) and COVID-19 vs normal (*N* = 138)

Classification	Sensitivity (%)	Specificity (%)	PPV (%)	NPV (%)	Accuracy (%)	AUC
COVID-19 vs bacterial pneumonia	95.35	97.44	97.62	95.00	96.34	0.98
COVID-19 vs non-COVID-19 viral pneumonia	97.44	97.67	97.44	97.67	97.56	0.98
COVID-19 vs normal	100	100	100	100	100	1.00

Table 5 shows the results of the multi-class classification using the XGB-L classifier. For multi-class classification problem, the average accuracy for classification of all four classes is used to measures the performance of the classifier (i.e., combined accuracy and AUC). Multi-class classification was able to classify COVID-19 amongst the four groups, with a combined AUC of 0.87 and accuracy of 79.52%. While with the top two ranked features, the combined AUC of 0.82 and accuracy of 66.27% was obtained. Sensitivity, specificity, positive predictive value, and negative predictive value were similarly high. As reflected in Tables 1, 2, 3 and 4, the two-class classification performance (i.e., COVID-19 vs normal, COVID-19 vs bacterial pneumonia, COVID-19 vs viral pneumonia) in terms of sensitivity and PPV was higher than 95%, while these measures using multi-class (COVID-19 vs normal vs bacterial vs viral pneumonia) could achieve performance greater than 74% and 83% to detect COVID-19, respectively.

**Table 5:** Multi-class classification using XGB-linear with texture + morphological features

Dataset	Sensitivity	Specificity	PPV	NPV	Combined accuracy	Combined AUC
XGB-linear						
Bacterial pneumonia	74.42%	86.18%	65.31%	90.60%	79.52%	0.87
COVID-19	74.49%	95.28%	83.78%	93.80%		
Normal	95.12	100	100	98.43		
Viral pneumonia	67.77%	91.06%	73.17%	89.60%		

Feature ranking algorithms are mostly used for ranking features independently without using any supervised or unsupervised learning algorithm. A specific method is used for feature ranking in which each feature is assigned a scoring value, then selection of features will be made purely on the basis of these scoring values [39]. The finally selected distinct and stable features can be ranked according to these scores and redundant features can be eliminated for further classification. We first extracted first extracted texture features based on GLCM and morphological features from COVID-19, normal, viral and bacterial pneumonia CXR images and then ranked them based on empirical receiver-operating characteristic curve (EROC) and random classifier slop [40], which ranks features based on the class separability criteria of the area between EROC and random classifier slope. The ranked features show the features importance based on their ranking which can be helpful for distinguish these different classes for improving the detection performance and decision making by the radiologists.

**Fig. 1:**
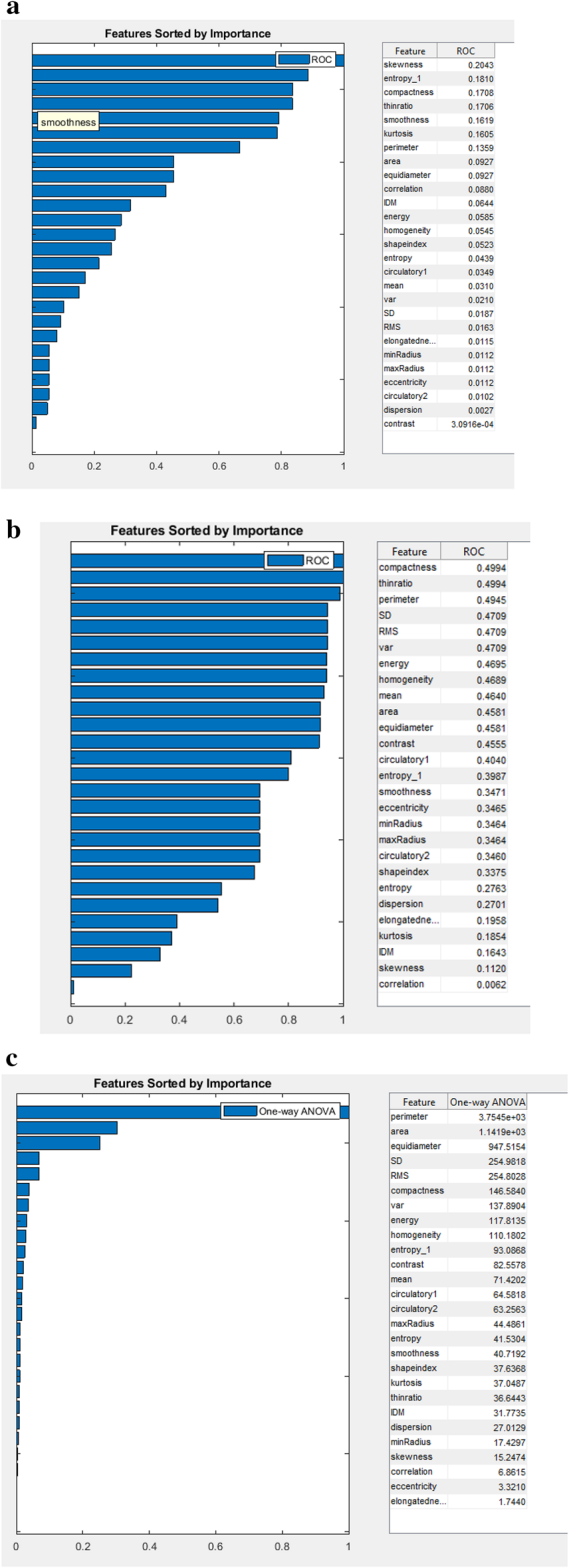
Ranking parameters: **a** COVID vs bacterial infection, **b** COVID-19 vs normal, and **c** multi-class feature ranking

Figure 1 shows the ranking features of COVID-19 vs bacterial infection, COVID-19 vs normal, and their multi-class features. The top four features from COVID-19 vs bacterial CXR based on AUC were: skewness, entropy, compactness, and thin ratio. The top four features from COVID-19 vs normal CXR based on AUC were: compactness, thin ratio, perimeter, and standard deviation. The top feature from the multi-class was by far perimeter.

## Discussion

We employed an automated supervised learning AI classification of texture and morphological-based features on portable CXRs to distinguish COVID-19 lung infections from normal, and other lung infections. The major finding was that the multi-class classification was able to accurately identify COVID-19 from amongst the four groups with a combined AUC of 0.87 and accuracy of 79.52%.

The hallmarks of COVID-19 lung infection on CXR are bilateral and peripheral hazy lung opacities and air space consolidation [15]. These features of COVID-19 lung infection likely stood out compared to other pneumonia, giving rise to distinguishable texture features. Our AI algorithm was able to distinguish COVID-19 vs normal CXR with 100% accuracy, COVID-19 vs bacterial pneumonia with 96.34% accuracy, and COVID-19 vs non-COVID-19 viral infection with 92.68% accuracy. These findings suggest that it is trivial to distinguish COVID-19 from normal CXR and the two viral infections were more similar than bacterial infection.

With the multi-class classification, all performance measures dropped significantly (except normal CXR) as expected. Nonetheless, the combined AUC and accuracy remained high. These findings are encouraging and suggest that the multi-class classification is able to distinguish COVID-19 lung infection from other similar lung infections.

The top four features from COVID-19 vs bacterial infection were skewness, entropy, compactness, and thin ratio. The top four features from COVID-19 vs normal were: compactness, thin ratio, perimeter, and standard deviation. The top feature from the multi-class was perimeter. Perimeter is the total count of pixels at the boundary of an image. It showed that the perimeter of COVID-19 lung CXRs differed significantly from other bacterial and viral infections as well as normal lung X-rays. These results together suggest that perimeter is a key distinguishable feature, consistent with a key observation that COVID-19 lung infection tends to be more peripheral and lateral together the boundaries of the lung.

A few studies have reported CNN analysis of CXR and CT for classification of COVID-19 [41–45]. Li et al. performed a retrospective multi-center study using a deep-learning model to extract visual features from chest CT to distinguish COVID-19 from community acquired pneumonia (CAP) and non-pneumonia CT with a sensitivity of 90%, specificity 95%, and AUC 0.96 (*p* value < 0.001) [41]. Hurt et al. performed a retrospective study using a U-net (CNN), to predict pixel-wise probability maps for pneumonia only from a public dataset that comprised of 22,000 radiographs. For their classification of pneumonia, the area under the receiver-operator characteristic curve was 0.854 with a sensitivity of 82.8% and specificity of 72.6 [46]. Wang et al. developed a deep CNN to detect COVID-19 cases from non-COVID CXR. This study used interpretable AI to visualize the location of the abnormality and was able to distinguish COVID-19 from non-COVID-19 viral infection, bacterial infection, and normal with a sensitivity of 81.9%, 93.1%, and 73.9%, respectively, with an overall accuracy of 83.5% [38]. Gozes et al. developed a deep-learning algorithm to analyze CT images to detect COVID-19 patients from non-COVID-19 cases with 0.996 AUC (95% CI 0.989–1.00), 98.2% sensitivity and 92.2% specificity [43]. Apostolopoulos and Mpesiana [45] used deep learning with a transfer learning approach to extract features from X-rays to distinguish between COVID-19 and bacterial pneumonia, viral pneumonia, and normal with a sensitivity of 98.66%, specificity of 96.46%, and accuracy of 96.78%. Overall, most of these studies used two-class comparison (i.e., pneumonia vs COVID-19, or pneumonia vs normal) mostly on CT which is less suitable for contagious diseases. In these previous studies, two-class prediction performance was computed and yielded fine results but could not achieve the highest performance as compared to our approach. The aim of this research was to improve the prediction performance by extracting texture and morphological features from CXR images. As the machine-learning performance is still a challenging task to extract the most relevant and appropriate features by the researchers. The results reveal that features extracted using our approach contain the most pertinent and appropriate hidden information present in the COVID-19 lung infection which improved the two-class and multi-class classification. These features are then used as input to the robust machine-learning classifiers. The results obtained outperformed than these previously traditional methods.

There are several limitations of this study. This is a retrospective study with a small COVID-19 sample size. Portable CXR is sensitive but not specific as the phenotypes of different lung infections are similar on CXR. We used only four classes (disease types). Future studies should expand to include additional lung disorders.

## Conclusion

In conclusion, deep learning of texture and morphological-based features accurately distinguish CXR of COVID-19 patients from normal subjects and patients with bacterial and non-COVID-19 viral pneumonia. This approach can be used to improve workflow, facilitate in early detection and diagnosis of COVID-19, effective triage of patients with or without the infectious disease, and provide efficient tracking of disease progression.

### Limitation and future directions

This study is specifically aimed to extract the texture features and apply the machine-learning algorithms to predict the COVID-19 from multi-class. The texture features correctly predict the COVID-19 from multi-class; however, in future, we will employ and optimize the deep convolutional neural network models including ResNet101, GoogleNet, AlexNet, Inception-V3 and use will use some other modalities, clinical profiles and bigger datasets.

## Methods

### Dataset

**Fig. 2:**
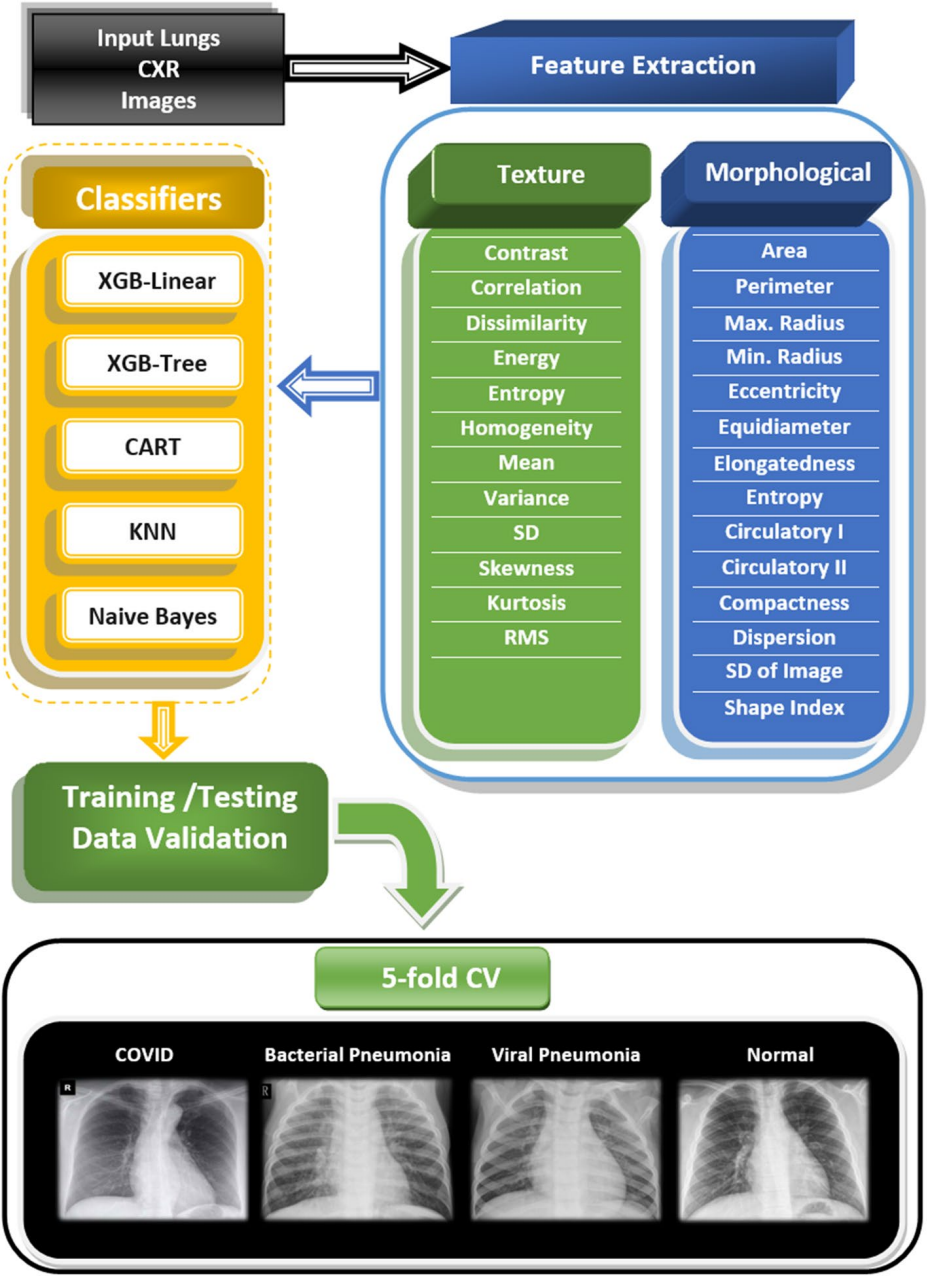
Flow of data and analysis

In this study, we used publicly available data of COVID-19 and non-COVID and normal chest CXR images. The COVID-19 images were downloaded from https://github.com/ieee8023/covid-chestxray-dataset [47] on Mar 31, 2020. The original download contained 250 scans of COVID-19 and SARS of CT and CXR taken in multiple directions. Two board-certified chest radiologists (one with 20 + years of experience) and one 2nd year radiology resident evaluated the images for quality and relevance. Only CXR from COVID-19 taken at anterior–posterior (AP) direction was included in this study, resulting in a final sample size of 130. The other dataset was taken from the Kaggle chest X-ray image (pneumonia) dataset (https://www.kaggle.com/paultimothymooney/chest-xray-pneumonia) [42]. Although the Kaggle database has a large sample size, we randomly selected a sample size comparable to that of COVID-19. The sample chosen for the bacterial pneumonia, non-COVID-19 viral pneumonia, and normal CXR were 145, 145, and 138, respectively. We first split the dataset into training and testing data with a 70% and 30% ratio using a stratified sampling method. Then for feature selection, we only used the training data instead of the whole dataset. Figure 2 below outlines the workflow and steps used in this study.

Figure 2 outlines the workflow with the initial input of lung CXRs going through feature extraction for texture + morphological analysis followed by the AI classifiers to determine the sensitivity, specificity, PPV, NPV, accuracy, and AUC of the four groups of interest (COVID-19, bacterial and viral pneumonia, and normal). These calculations are further outputted for data validation with fivefold cross-validation technique. Finally, data are statistically analyzed for significance using MATLAB 2018b and RStudio 1.2.5001.

#### Texture features

The texture features are estimated from the Grey-level Co-occurrence Matrix (GLCM) covering the pixel (image) spatial correlation. Each GLCM input image $\left( {u,v} \right){\text{th}}$ defines how often pixels with intensity value $u$ co-occur in a defined connection with pixels with intensity value $v$. We extracted second-order features consisting of contrast, correlation, mean, entropy, energy, variance, inverse different moment, standard deviation, smoothness, root mean square, skewness, kurtosis, and homogeneity previously used in [48–54].

#### Morphological features

Morphological feature plays an important role in the detection of malignant tissues. Morphological features convert image morphology into a set of quantitative values that can be used for classification [55]. Morphological feature-extracting method (MFEM) is a nonlinear filtering process and its basic purpose is to search and find valuable information from an image and transform it morphologically according to the requirements for segmentation [56] and so on. The MFEM takes binary cluster as an input and finds the associated components in the clusters having an area greater than a certain threshold. There are several features that can be extracted from an image and area can be calculated from the number of pixels of an image. Area and perimeter combined helps to calculate the values of other different morphology features. The following formulas in [50] can be used to calculate the values of morphological features.

#### Classification

We applied and compared five supervised machine-learning classification algorithms: XG boosting linear (XGB-L), XG boosting tree (XGB-tree), classification and regression tree (CART), k-nearest neighbor (KNN) and Naïve Bayes (NB). We used XGB ensemble methods in this study. In machine learning, ensemble is the collection of multiple models and is one of the self-efficient methods as compared to other basic models. Ensemble technique combines different hypothesis to hopefully provide best hypothesis. Basically, this method is used for obtaining a strong learner with the help of combination of weak learners Experimentally, ensembles methods provide more accurate results even there is considerable diversity between the models. Boosting is a most common types of ensemble method that works by discovering many weak classification rules using subset of the training examples simply by sampling again and again from the distribution.

#### XGBoost algorithms

Chen and Guestrin proposed XGBoost a gradable machine-learning system in 2016 [57]. This system was most popular and became the standard system when it was employed in the field of machine learning in 2015 and it provides us with better performance in supervised machine learning. The Gradient boosting model is the original model of XGBoost, which combine and relates a weak base with stronger learning models in an iterative manner [58]. In this study, we used XGBoost linear and tree with following optimization parameters.

We used the following parameter of each model in this study. For XGB-linear we initialized the parameters as lambda = 0, alpha = 0 and eta = 0.3, where lambda and alpha are the regularization term on weights and eta is the learning rate. For XGB-Tree, we initialized the parameters with maximum depth of tree i.e., max-depth = 30, learning rate eta = 0.3, maximum loss reduction i.e., gamma = 1, minimum child weight = 1, subsample = 1. The nearest neighbor k = 5 was used. For CART, we initialized parameters with minsplit = 20, complexity parameter, i.e., cp = 0.01, maximum depth = 30. For Naïve Bayes, we initialized the parameters with search method = grid, laplace = 0, and adjust = 1.

#### Classification and regression tree (CART)

A CART is a predictive algorithm used in the machine learning to explain how the target variable values can be predicted based on the other values. It is a decision tree where each fork is a split in a predictor variable and each node at the end has a prediction for the target variable. Decision tree (DT) algorithm was first proposed by Breiman in 1984 [59], is a learning algorithm or predictive model or decision support tool of Machine Learning and Data Mining for the large size of input data, which predict the target value or class label based on several input variables. In decision tree, the classifier compares and checks the similarities in the dataset and ranked it into distinct classes. Wang et al. [60] used DTs for classifying the data based on choice of an attribute which maximizes and fix the data division. Until the conclusion criteria and condition is met, the attributes of datasets are split into several classes. DT algorithm is constructed mathematically as:

$$ \overline{X} = \{ X_{1} ,X_{2} ,X_{3} , \ldots ,X_{m} \}^{{\text{T }}} , $$

$$ X_{i} = \left\{ {x_{1} ,x_{2} ,x_{3} , \ldots ,x_{ij} , \ldots ,x_{in} } \right\}, $$

$$ S = \left\{ {S_{1} ,S_{2} , \ldots ,S_{i} , \ldots ,S_{m} } \right\}. $$

Here the number of observations is denoted by m in the above equations, n represent number of independent variables, S is the m-dimension vector spaces of the variable forecasted from $\overline{X}$ in the above equation. $X_{i}$ is the *i*th module of *n*-dimension autonomous variables $x_{i1} ,x_{i2} ,x_{i3} , \ldots ,x_{in}$ are autonomous variable of pattern vector $X_{i}$ and T is the transpose symbol.

The purpose of DTs is to forecast the observations of $\overline{X}$. From $\overline{X}$, several DTs can be developed by different accuracy level; although, the best and optimum DT construction is a challenge due to the exploring space has enormous and large dimension. For DT, appropriate fitting algorithms can be developed which reflect the trade-off between complexity and accuracy. For partition of the dataset $\overline{X}$, there are several sequences of local optimum decision about the feature parameters are used using the Decision Tree strategies. Optimal DT, $T_{k0}$ is developed according to a subsequent optimization problem:

$$ \hat{R}\left( {T_{k0} } \right) = \min \left\{ {\hat{R}\left( {T_{k0} } \right)} \right\}, k = 1,2,3, \ldots ,K, $$

$$ \hat{R}\left( T \right) = \mathop \sum \limits_{t \in T}^{k} \left\{ {r\left( t \right)p\left( t \right)} \right\} . $$

In the above equation, $\hat{R}\left( T \right)$ represents an error level during the misclassification of tree $T_{k}$, $T_{k0}$ represented the optimal DT that minimizes an error of misclassification in the binary tree, T represent a binary tree $\in \left\{ {T_{1} ,T_{2} , \ldots ,T_{k} ,t_{1} } \right\}$, the index of tree is represented by k, tree node with t, root node by t1, resubstituting an error by *r*(*t*) which misclassify node t, probability that any case drop into node t is represented with *p*(*t*). The left and right sets of partition of sub trees are denoted by $T^{L} \; {\text{and}}\; T^{R}$. The result of feature plan portioning the tree *T* is formed.

#### Naïve Bayes (NB)

The NB [61] algorithm is based on Bayesian theorem [62] and it is suitable for higher dimensionality problems. This algorithm is also suitable for several independent variables whether they are categorical or continuous. Moreover, this algorithm can be the better choice for the average higher classification performance problem and have minimal computational time to construct the model. Naïve Bayes classification algorithm was introduced by Wallace and Masteller in 1963. Naïve Bayes relates with a family of probabilistic classifier and established on Bayes theorem containing compact hypothesis of independence among several features. Naïve Bayes is most ubiquitous classifier used for clustering in Machine Learning since 1960. Classification probabilities are able to compute using Naïve Bayes method in machine learning. Naïve Bayes is utmost general classification techniques due to highest performance than the other algorithm such as decision tree (DT), C-means (CM) and SVM. Bayes decision law is used to find the predictable misclassification ratio whereas assuming that true classification opportunity of an object belongs to every class is identified. NB techniques were greatly biased because its probability computation errors are large. To overcome this task, the solution is to reduce the probability valuation errors by Naïve Bayes method. Conversely, dropping probability computation errors did not provide the guarantee for achieving better results in classification performance and usually make it poorest because of its different bias-variance decomposition among classification errors and probability computation error [63]. Naïve Bayes is widely used in present advance developments [64–67] due to its better performance [68]. Naïve Bayes techniques need a large number of parameters during learning system or process. The maximum possibility of Naïve Bayes function is used for parameter approximation. NB represents conditional probability classifier which can be calculated using Bayes theorem: problem instance which is to be classified, described by a vector $Y = \left\{ {Y_{1} , Y_{2} , Y_{3} , \ldots ,Y_{n} } \right\}$ shows n features spaces, conditional probability can be written as:

$$ S(N_{k} |Y_{1} , Y_{2} , Y_{3} , \ldots Y_{n} ). $$

For each class $N_{k}$ or each promising output, statistically Bayes theorem can be written as:

$$ S\left( {N_{k} |Y} \right) = \frac{{S\left( {N_{k} } \right)S\left( {Y|N_{k} } \right)}}{S\left( Y \right)}. $$

Here, $S\left( {N_{k} {|}Y} \right)$ represents the posterior probability while $S\left( {N_{k} } \right)$ represents the preceding probability, $S\left( {Y|N_{k} } \right)$ represents the likelihood and $S\left( Y \right)$ represents the evidence. NB is represented mathematically as:

$$ S\left( {N_{k} |Y_{1} , Y_{2} , Y_{3} , \ldots ,Y_{n} } \right) = \frac{1}{T}S\left( {N_{k} } \right)\mathop \prod \limits_{i = 1}^{n} S(Y_{i} |N_{k} ). $$

Here $T = S\left( y \right)$ is scaling factor which is depends upon $(Y_{1} , Y_{2} , Y_{3} , \ldots ,Y_{n} )$, $S\left( {N_{k} } \right)$ is a parameter used for the calculation of marginal probability and conditional probability for each attribute or instances is represented by $S(Y_{i} |N_{k} )$. Naïve Bayes become most sensitive in the presence of correlated attributes. The existence of extremely redundant or correlated objects or features can bias the decision taken by Naïve Bayes classifier [67].

#### K-nearest neighbor (KNN)

KNN is most widely used algorithm in the field of machine learning, pattern recognition and many other areas. Zhang [69] used KNN for classification problems. This algorithm is also known as instance based (lazy learning) algorithm. A model or classifier is not immediately built but all training data samples are saved and waited until new observations need to be classified. This characteristic of lazy learning algorithm makes it better than eager learning, that construct classifier before new observation needs to be classified. Schwenker and Trentin [70] investigated that this algorithm is also more significant when dynamic data are required to be changed and updated more rapidly. KNN with different distance metrics were employed. KNN algorithm works according to the following steps using Euclidean distance formula.

Step I: To train the system, provide the feature space to KNN.

Step II: Measure distance using Euclidean distance formula:

$$ d\left( {x_{i} , y_{i} } \right) = \mathop \sum \limits_{i = 1}^{n} \sqrt {(x_{i - } y_{i} )^{2} } . $$

Step III: Sort the values calculated using Euclidean distance using $d_{i} \le d_{i} + 1, \;{\text{where}}\; i = 1,2,3, \ldots ,k$.

Step IV: Apply means or voting according to the nature of data.

Step V: Value of *K* (i.e., number of nearest Neighbors) depends upon the volume and nature of data provided to KNN. For large data, the value of *k* is kept as large, whereas for small data the value of *k* is also kept small.

In this study, these classification algorithms were performed using RStudio with typical default parameters for each of the classifiers (XGB-L, GXB-tree, CART, KNN, NB) with a fivefold cross-validation. As we divided our dataset into train and test sets, so while training a classifier on train data we used the K-fold cross-validation technique, which shuffles the data and splits it into *k* number of folds (groups). In general, K-fold validation is performed by taking one group as the test data set, and the other *k* − 1 groups as the training data, fitting and evaluating a model, and recording the chosen score on each fold. As we used fivefold cross-validation, so the train set is equally divided into five parts from which one is used as validation and the other four used for training of classifier on each fold.

#### Performance evaluation measures

The performance was evaluated with the following parameters.

##### Sensitivity

The sensitivity measure also known as TPR or recall is used to test the proportion of people who test positive for the disease among those who have the disease. Mathematically, it is expressed as:

$$ {\text{Sensitivity}} = \frac{{\mathop \sum \nolimits {\text{True}}\;{\text{ positive}}}}{{\mathop \sum \nolimits {\text{Condition}}\; {\text{positive}}}}, $$

$$ {\text{Sensitivity}} = \frac{{{\text{TP}}}}{{{\text{TP}} + {\text{FN}}}}, $$

i.e., the probability of positive test given that patient has disease.

##### Specificity

The TNR measure also known as specificity is the proportion of negatives that are correctly identified. Mathematically, it is expressed as:

$$ {\text{Specificity}} = \frac{{\mathop \sum \nolimits {\text{True }}\;{\text{negative}}}}{{\mathop \sum \nolimits {\text{Condition}}\;{\text{negative}}}}, $$

$$ {\text{Specificity}} = \frac{{{\text{TN}}}}{{{\text{TN}} + {\text{FP}}}}, $$

i.e., probability of a negative test given that patient is well.

##### Positive predictive value (PPV)

PPV is mathematically expressed as:

$$ {\text{PPV}} = \frac{{\sum {{\text{True}}\;{\text{positive}}} }}{{\sum {{\text{Predicted}}\;{\text{condition}}\;{\text{positive}}} }}, $$

$$ {\text{PPV}} = \frac{{{\text{TP}}}}{{{\text{TP}} + {\text{FP}}}}, $$

where TP denotes that the test makes a positive prediction and subject has a positive result under gold standard while FP is the event that test make a positive perdition and subject make a negative result.

##### Negative predictive value (NPV)

NPV can be computed as:

$$ {\text{NPV}} = \frac{{\sum {{\text{True }}\;{\text{negative}}} }}{{\sum {{\text{Predicted}}\; {\text{condition}}\;{\text{negative}}} }}, $$

$$ {\text{NPV}} = \frac{{{\text{TN}}}}{{{\text{TN}} + {\text{FN}}}}, $$

where TN indicates that test make negative prediction and subject has also negative result, while FN indicate that test make negative prediction and subject has positive result.

##### Accuracy

The total accuracy is computed as:

$$ {\text{Accuracy}} = \frac{{{\text{TP}} + {\text{TN}}}}{{{\text{TP}} + {\text{FP}} + {\text{FN}} + {\text{TN}}}}. $$

#### Receiver-operating characteristic (ROC) curve

Based on sensitivity, i.e., true-positive rate (TPR) and specificity, i.e., false-positive rate (FPR) values of COVID-19 and non-COVID subjects. The mean values for COVID-19 subjects are classified as 0 and for non-COVID subjects are classified as 1. Then obtained vector is passed through ROC function, which plots each value against sensitivity and specificity values. ROC is considered as one of the standard methods for computation and graphical representation of the performance of a classifier. ROC plots FPR against *x*-axis and TPR against *y*-axis, while part of a square unit is represented by area under the curve (AUC). The value of AUC lies between 0 and 1 where AUC > 0.5 indicates the separation. Higher area under the curve represents the better and improved diagnostic system [71]. The number of correct positive cases divided by the total number of positive cases represents TPR. While the number of negative cases predicted as positive cases divided by the total number of negative cases represent FPR [72].

#### Training/testing data formulation

The Jack-knife fivefold cross-validation (CV) technique was applied for the training and testing of data formulation and parameter optimization. It is one of the most well known, commonly practiced, and successfully used methods for validating the accuracy of a classifier using fivefold CV. The data are divided into fivefold in training, the fourfold participate, and classes of the samples for remaining folds are classified based on the training performed on fourfold. For the trained models, the test samples in the test fold are purely unseen. The entire process is repeated five times and each class sample is classified accordingly. Finally, the unseen samples classified labels that are to be used for determining the classification accuracy. This process is repeated for each combination of each systems’ parameters and the classification performance have been reported for the samples as depicted in the Tables 1, 2, 3 and 4.

### Statistical analysis and performance measures

Analyses examining differences in outcomes used unpaired two-tailed *t* tests with unequal variance. Receiver-operating characteristic (ROC) curve analysis was performed with COVID-19, normal, bacterial, and non-COVID-19 viral pneumonia as ground truth. The performance was evaluated by standard ROC analysis, including sensitivity, specificity, positive predictive value (PPV), negative predictive value (NPV), accuracy, area under the receiver-operating curve (AUC) with 95% confidence interval, and significance with the *P* value. AUC with lower and upper bounds and accuracy were tabulated. MATLAB (R2018b, MathWorks, Natick, MA) and RStudio 1.2.5001 were used for statistical analysis.

## Abbreviations

MERS: Middle East respiratory syndrome
WHO: World Health Organization
ARDS: Acute respiratory distress syndrome
RT-PCR: Polymerase chain reaction
CT: Computed tomography
CXR: Chest X-ray
CAD: Computer-aided diagnostic
BNs: Bayesian networks
SVM: Support vector machine
ANNs: Artificial neural networks
kNN: K-nearest neighbors
DTs: Adaboost, decision trees
SIFT: Scale-invariant Fourier transform
EFDs: Elliptic Fourier descriptors
ML: Machine learning
DCNN: Deep convolutional neural network
CV: Cross-validation
ROC: Receiver-operating characteristic
PPV: Positive predictive value
NPV: Negative predictive value

## Availability of supporting data and materials

These data are already available via https://github.com/ieee8023/covid-chestxray-dataset.
